# From natural induction to artificial regulation: a review on the mechanisms and techniques of flowering in pineapple

**DOI:** 10.3389/fpls.2025.1751953

**Published:** 2026-01-22

**Authors:** Linpan Chen, Miaolin Zhang, Xiumei Zhang, Aiqi Tan, Fangyao Zhong, Junjun He, Qingsong Wu, Yanli Yao, Chuanling Li

**Affiliations:** 1 Key Laboratory of Postharvest Physiology and Technology of Tropical Horticultural Products of Hainan Province, International Joint Research Center for the Pineapple Cultivation Techniques, South Subtropical Crops Research Institute, Chinese Academy of Tropical Agricultural Sciences, Zhanjiang, Guangdong, China; 2 Sanya Research Institute of Chinese Academy of Tropical Agricultural Sciences, Sanya, Hainan, China; 3 Lingnan Normal University, Zhanjiang, Guangdong, China

**Keywords:** ethylene, floral bud differentiation, flower morphological characteristics, flowering regulation, pineapple

## Abstract

Flowering is a pivotal developmental transition in the life cycle of plants, and the precise timing of this process is crucial for successful reproduction. The flowering mechanism of the pineapple is influenced by a combination of genetic factors, environmental conditions and cultivation methods. Once pineapple plants have reached a certain number of leaves, the timing of floral bud differentiation can be regulated by applying plant growth regulators. This facilitates staggered fruit production and enables a balanced year-round supply. The timing, quantity and quality of floral bud differentiation directly affect pineapple fruit quality and yield, and also significantly impact the economic and social benefits of the pineapple industry. This paper provides a systematic review of the morphological characteristics of flowers, the patterns of floral bud differentiation, the mechanisms underlying natural and induced flowering, and the key factors influencing flowering in pineapples. This review establishes a theoretical foundation for regulating fruiting periods and optimising high-quality, high-efficiency cultivation practices.

## 1 Introduction

Among global tropical cash crops, pineapple (*Ananas comosus* (L.) Merr) has become a core crop within the agricultural industrial structures of numerous tropical and subtropical regions, owing to its rich nutritional value, extensive processing applications, and substantial economic returns (Mohd Ali et al., 2020; Li et al., 2024). From field cultivation to market distribution, pineapple production efficiency is highly dependent on the critical developmental stage of flowering. The timing, quantity, and quality of flower bud differentiation directly determine the subsequent fruit maturation cycle, commercial characteristics, and yield levels, thereby influencing the stability of returns across the entire industry chain (Sun et al., 2019; Liu et al., 2021). Pineapple flowering is inherently constrained by its genetic background, dynamically influenced by environmental factors such as temperature and photoperiod, and amenable to artificial intervention through cultivation practices. The interplay of these elements collectively constitutes the regulatory network governing pineapple flowering (Wang et al., 2017).

In traditional cultivation practices, natural flowering is the primary method of pineapple propagation, but this process has significant limitations (Van de Poel et al., 2009). For example, natural flowering results in inconsistent harvest time, which prevents centralised market release and increases harvesting costs, meanwhile off-season natural flowering increases post-harvest costs and degrades fruit quality (Van de Poel et al., 2009; Liu et al., 2021). Consequently, chemical agents such as ethephon and calcium carbide are routinely used in pineapple production to regulate flowering time precisely, enabling ‘batch flowering and off-season fruiting’ and helping to balance market supply and demand (Bartholomew and Sanewski, 2018; Lin et al., 2023). Nevertheless, there are still numerous challenges associated with current artificial flowering induction techniques, such as significant variations in sensitivity to inducers across different cultivars, and the risk of reduced fruit quality due to improper application (Liu et al., 2009; Zhang et al., 2018; Pu et al., 2024).

Significant advances have been made over the past several decades in our understanding of the flowering mechanism of pineapples (Bartholomew, 1997; Liu et al., 2011; Zhang et al., 2021). Research has confirmed that ethylene is the key signalling molecule that triggers pineapple flowering (Zhang et al., 2018). It regulates this process through synergistic or antagonistic interactions with other hormones, such as abscisic acid (ABA), gibberellin (GA_3_) and cytokinins (Achard et al., 2007; Espinosa et al., 2017; Yow et al., 2023). Furthermore, the *AP2/ERF*, *MADS-box* and *PEBP* gene families have been identified as mediators of the flowering response in pineapples to environmental and hormonal signals (Zhang et al., 2020, 2021; Pan et al., 2022; Zhang et al., 2023).

Despite the substantial data accumulated through existing research, a systematic integration of the morphological, physiological, molecular biological, and cultivation techniques pertaining to pineapple flowering remains elusive. Consequently, this paper provides a systematic review of the morphological characteristics of pineapple floral organs, the pattern of floral bud differentiation, the regulatory mechanisms governing natural and induced flowering, key influencing factors such as temperature, photoperiod and endogenous hormones, and the current status and limitations of techniques for inducing and inhibiting flowering. It aims to provide theoretical underpinnings for optimising flowering regulation strategies in pineapples, enhancing management efficiency during the fruiting period, and advancing high-quality, efficient production within the global pineapple industry.

## 2 Morphological characteristics of pineapple flowers

**Figure 1:**
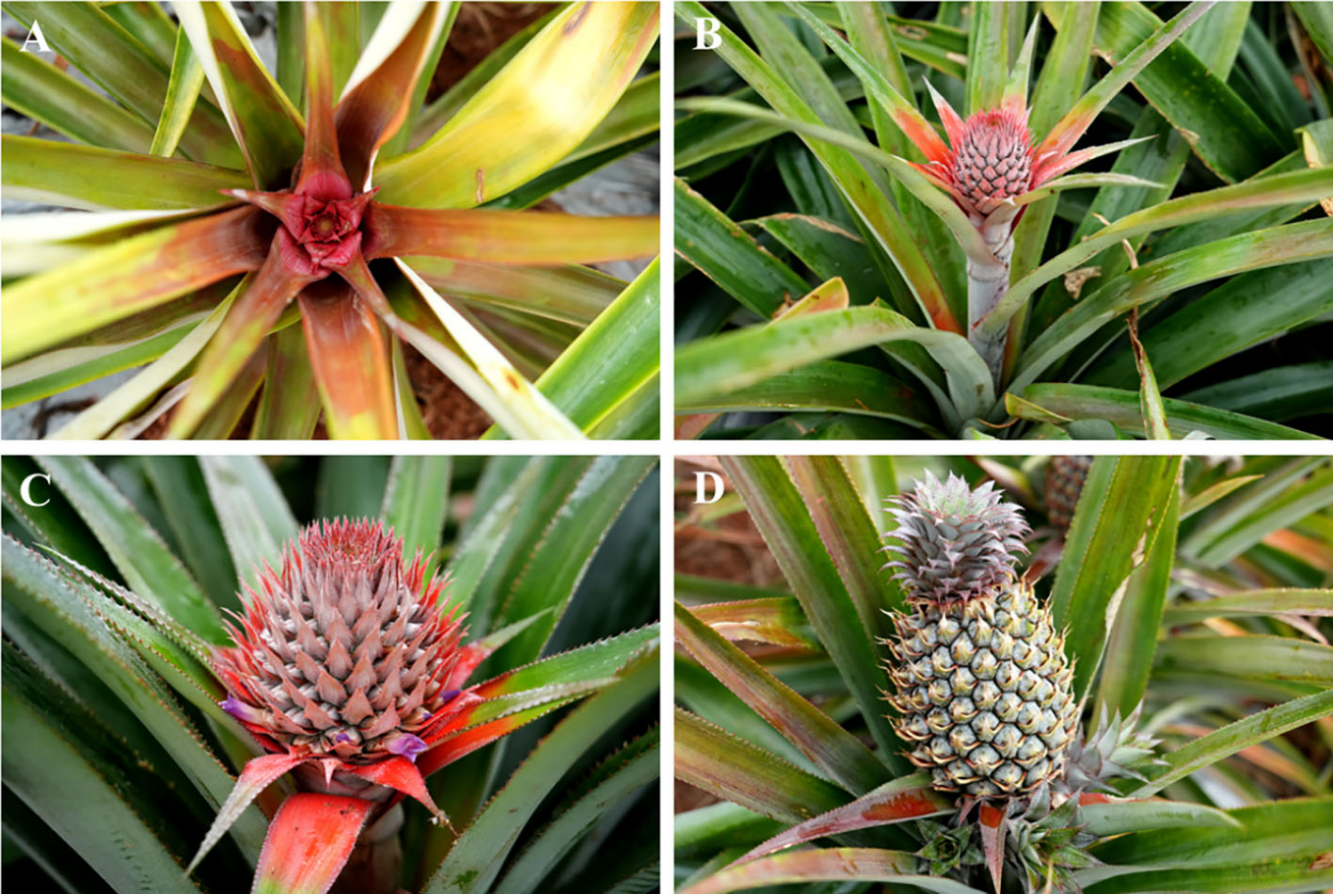
The developmental process of pineapple flowers. **(A)** The base of the pineapple leaves turns red. **(B)** Pineapple inflorescence emergence. **(C)** Flowering. **(D)** Developing into an aggregate fruit.

When the pineapple transitions from vegetative to reproductive growth, the apical meristem at the stem tip undergoes a sequential process: the leaf bases turn red, the inflorescence emerges, flowering occurs, and fruit sets, ultimately developing into an aggregate fruit (**Figures 1A–D**) (Xue et al., 2023; Li and Hu, 2024).

**Figure 2:**
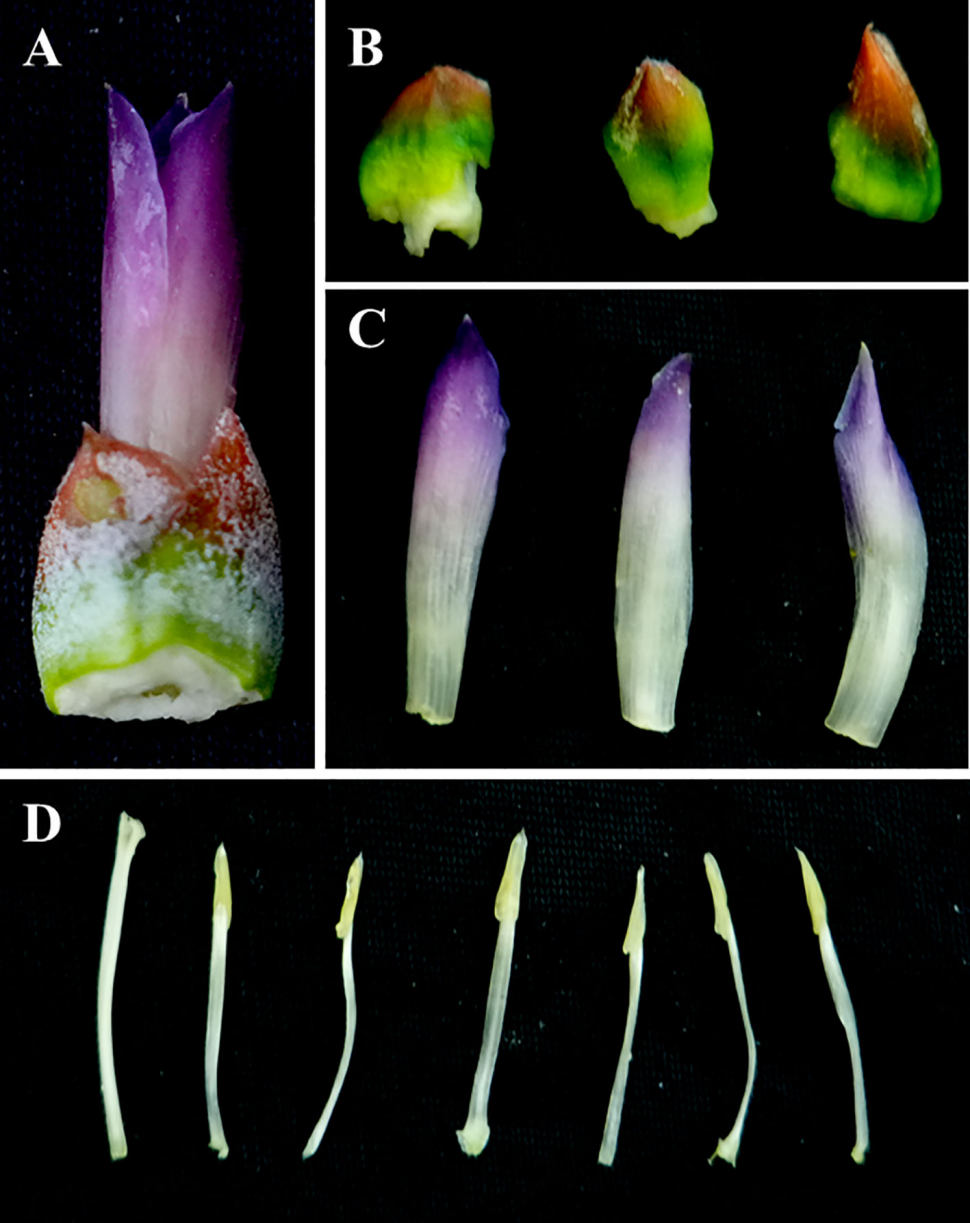
Structural characteristics of pineapple flowers. **(A)** Single flower bud. **(B)** Three fleshy bracts. **(C)** Three petals. **(D)** One pistil (leftmost) and six stamens.

The pineapple’s inflorescence is a terminal compound raceme with a fleshy central axis that serves as the core supporting structure. Around this axis are borne 60–200 sessile, perfect flowers (Lu et al., 2023). The entire inflorescence emerges naturally from the apical leaf cluster at the top of the stem and is protected at its base by 5–7 red bracts, which are usually red in colour, though occasionally green or pale yellow. These bracts serve a protective function (Lu et al., 2023). The pineapple’s solitary flower comprises three fleshy bracts and three petals, each approximately 2 cm long. These petals overlap to form a tubular structure. The petals are usually white at the base and purple red towards the top (Tan et al., 2015; Xue et al., 2023). Six stamens are arranged in two regular whorls and the pistil bears a three lobed stigma with an inferior, three locular ovary. Each locule contains 14–20 ovules arranged in two rows and enveloped externally by red bracts (see **Figures 2A–D**).

The flowering of pineapples exhibits distinct temporal characteristics. The basal florets open first and then progressively ascend upwards. The entire flowering period lasts 25–30 days (Xue et al., 2023). The peak flowering time of the florets is around 9:00 a.m. each day and each floret lasts approximately 24 hours from bud break to senescence (Xue et al., 2023). Under suitable temperatures, more florets open earlier, leading to earlier fruit ripening. Conversely, low temperatures with overcast and rainy conditions or high temperatures with drought are detrimental to flowering (Maruthasalam et al., 2010; Chen et al., 2016; Yow et al., 2022). The number of florets is closely correlated with fruit size. When plants are vigorous and well nourished, the number of florets increases, leading to higher yields (Tan et al., 2015; Liu et al., 2021). Furthermore, pineapples exhibit self-incompatibility, whereby pollination between flowers on the same plant, different flowers on the same plant, or different plants fails to produce seeds. This trait is advantageous for fresh consumption (Sun et al., 2019; Xue et al., 2023). However, cross pollination between different cultivars produces seeds, enabling breeders to conduct artificial pollination and develop new varieties through hybrid seed selection (Sun et al., 2019; Xue et al., 2023).

## 3 Pineapple floral bud differentiation

Floral bud differentiation is a pivotal stage in a plant’s life cycle, marking the transition from vegetative to reproductive growth. The timing, quantity and quality of this process are closely linked to a crop’s earliness and yield, which is of great importance for agricultural production (Liu et al., 2011; Kinoshita and Richter, 2020).

The timing of flower bud differentiation varies among pineapple cultivars across different regions (Sun et al., 2019). For example, in southern Taiwan, the period during which the flower buds of the ‘Red Skin’ pineapple differentiate commences around 20th of November and concludes by 20th of December, spanning approximately 40 days (Sun et al., 2019). For ‘Smooth Cayenne’ pineapples, the morphological differentiation phase commences later, starting on 5th of December and 20th of December respectively, concluding by 20th of January the following year, spanning 30–40 days (Sun et al., 2019). In the Zhanjiang region during 2008–2009, pineapple flower bud differentiation commenced from mid to late December, with the floral organ differentiation phase occurring from mid-January to mid-February (Liu et al., 2011; Sun et al., 2019).

Preliminary studies on the morphological differentiation of pineapple floral buds have been conducted both domestically and internationally. Kerns et al. (1936) analysed the relationship between the width of pineapple growth tissues and the morphology of flower bud differentiation using paraffin sectioning techniques. They discovered that the width of the meristematic tissue at the apical meristem changed before and after morphological differentiation of the flower buds. Bartholomew (1997) observed the differentiation process of ‘Smooth Cayenne’ pineapple flower buds following ethephon induced flowering using scanning electron microscopy. They found that the first structure to differentiate within the inflorescence was the involucral bract, which subsequently differentiated into three sepal primordia and one petal primordium. These then developed into six stamens and three carpels (Bartholomew, 1997).

**Figure 3:**
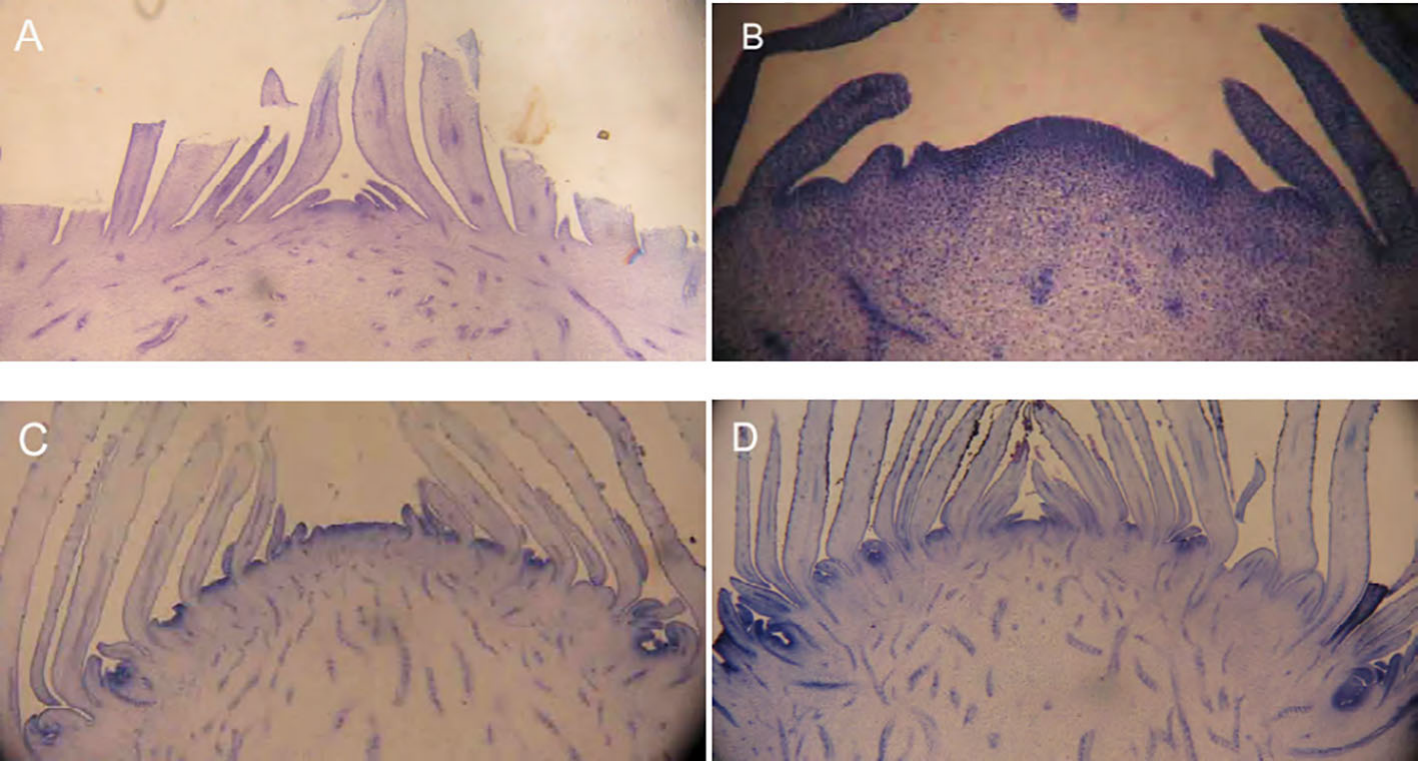
Tissue sections of pineapple floral buds (Adapted from Liu et al., 2011). **(A)** Undifferentiated stage. **(B)** Inflorescence differentiation stage. **(C)** Floral organ differentiation stage. **(D)** Crown bud formation stage.

In general, floral bud differentiation can be divided into four stages: ① Undifferentiated stage: The internodal tissues are tightly enclosed by leaves, and the growing point is flat and narrow (**Figure 3A**); ② Inflorescence differentiation stage: The cells of the stem apical meristem undergo vigorous division, the stem apex widens and protrudes upward, the formation of leaf primordia ceases, the development of inflorescence primordia initiates, and involucral bracts begin to form (**Figure 3B**); ③ Floral organ differentiation stage: Numerous small protrusions (the primordia of florets and inflorescences) form around the growing point; the primordia of bracteoles, sepals, perianths, stamens, and pistils differentiate sequentially from the outside to the inside in florets (**Figure 3C**); ④ Crown bud formation stage: The crown bud primordia form and extend upward (Liu et al., 2011; Sun et al., 2019) (**Figure 3D**).

## 4 Pineapple flowering

### 4.1 Modes of pineapple flowering

Pineapple flowering is mainly classified into two main types: natural flowering and induced flowering (Fassinou Hotegni et al., 2015; Wang et al., 2017; Paull et al., 2023). Natural flowering occurs when flower bud differentiation takes place in pineapple plants without the application of plant growth regulators, which is comprehensively influenced by plant weight, cultivar, and environmental conditions (Fassinou Hotegni et al., 2015; Sun et al., 2019). There are two scenarios of natural flowering: firstly, normal natural flowering, where the plants have reached a specific seedling age and have the required leaf count and plant weight for commercial fruit production (Sun et al., 2019; Liu et al., 2021); The second scenario is premature flowering, which occurs when plants flower ahead of schedule due to environmental stresses such as photoperiod, temperature or drought, before reaching the required leaf count and plant weight for commercial fruit production (Liu et al., 2011; Sun et al., 2019; Liu et al., 2021).

Naturally flowering pineapples that come into season outside of the usual period typically yield smaller fruits with scattered harvest periods, making concentrated sales unfeasible. This increases harvesting costs, which has a significant impact on the healthy development of the pineapple industry (Van de Poel et al., 2009).

Therefore, during the production process, when plants reach a certain seedling age, plant growth regulators are typically applied to release ethylene or acetylene, stimulating the plants to flower earlier and more uniformly. This practice is termed induced flowering (Fassinou Hotegni et al., 2014; Espinosa et al., 2017; Lin et al., 2023). Artificially regulating flowering timing not only addresses the drawbacks of natural flowering, but also enables staggered market release and year round pineapple supply. However, it should be noted that successful flowering induction remains challenging even with ethephon treatment if pineapple plants are too small (Van de Poel et al., 2009; Liu et al., 2011).

### 4.2 Factors affecting flowering in pineapples

#### 4.2.1 Temperature

As early as 1979, Friend’s research revealed that night time temperatures significantly influence pineapple flowering. For the ‘Smooth Cayenn’ cultivar, flowering occurred most rapidly at a night time temperature of 20°C. Flowering was slowest at night temperatures of 15°C or 25°C; at 30°C, pineapples failed to flower for three years (Friend and Lydon, 1979).

Temperature significantly influences the differentiation of pineapple floral buds (Huang and Lin, 2003; Min and Bartholomew, 1997; Maruthasalam et al., 2010; Yow et al., 2023). Maruthasalam et al. (2010) indicate that, following a brief cold treatment, the ethylene biomass within pineapple shoot tips doubles compared to the control group. Treating the shoot tips four times with 500 g of ice water can induce flowering.

Additionally, Yow et al. (2023) examined the impact of low temperature stress on pineapple flowering via transcriptome analysis, employing the early flowering tolerant cultivar ‘Dole-17’ and the flowering-prone cultivar ‘MD-2’ as research subjects. The results showed that, under low temperature conditions, genes involved in ethylene and abscisic acid (ABA) mediated pathways were expressed more significantly in ‘MD-2’. This may lead to early flowering due to hormone sensitivity. Meanwhile, flowering was delayed in ‘Dole-17’ due to the downregulation of ethylene responsive genes (*AP2/ERF*).

#### 4.2.2 Photoperiod

The transition from the vegetative to the reproductive phase (flowering) is a crucial developmental process for flowering plants, influencing their reproductive success (Creux and Harmer, 2019; Maple et al., 2024). The timing of pineapple flowering is regulated by environmental factors, such as changes in day length and light intensity (Shrestha et al., 2014; Cho et al., 2017; Shim and Jang, 2020). Optimal light intensity promotes the accumulation of photosynthetic products (such as carbohydrates), providing the energy and material basis for flower bud differentiation. However, excessive light may induce photoinhibition, damaging the photosynthetic system, or delay flowering by inducing the accumulation of stress hormones (such as abscisic acid). while insufficient light, due to inadequate photosynthetic products, impedes floral bud differentiation, delays flowering, or even prevents flowering altogether (Luo et al., 2022; Gendron and Staiger, 2023; Liu et al., 2023).

During pineapple production, providing 90% shading to plants significantly delays flowering. Combining shading with a 1% urea treatment further enhances this delay effect (Lin et al., 2015). Both shading and urea treatment reduce the carbon-to-nitrogen ratio by increasing leaf nitrogen concentration, thereby inhibiting leaf starch accumulation and delaying flowering (Lin et al., 2015). Prolonged shading adversely affects fruit size and quality. However, following shortterm shading, exposing plants to light recovery followed by calcium carbide treatment for flowering induction resulted in fruit quality showing no significant difference compared to the control (Lin et al., 2015).

#### 4.2.3 Endogenous hormones

During the transition from vegetative to reproductive growth in pineapples, plant hormones play a central regulatory role, with complex synergistic or antagonistic interactions occurring between different hormones. Furthermore, this regulatory mechanism exhibits distinct characteristics depending on the pineapple cultivar (Ogawara et al., 2003; Achard et al., 2007; Wuriyanghan et al., 2009; Wang et al., 2013).

Ethylene is a plant hormone that regulates plant growth and development, as well as responses to biotic and abiotic stresses (Kazan, 2015; Liu and Chen, 2021; Hausinger et al., 2023; Shekhawat et al., 2023). Ethylene is the key hormone triggering flowering in pineapples (Espinosa et al., 2017; Wang et al., 2017). Under natural conditions, reproductive development in pineapples is induced by environmental signals such as photoperiod and growth temperature, ultimately mediated by an ethylene surge or increased ethylene sensitivity in the apical meristem (Chang et al., 2011; Bartholomew and Sanewski, 2018). In commercial production, artificial application of ethylene or ethylene releasing chemicals, such as ethephon or calcium carbide, constitutes a conventional method for inducing synchronised flowering in pineapples (Dass et al., 1975, 1976; Liu et al., 2011; Sun et al., 2019). Following ethephon treatment, signs of differentiation appear within 48–72 hours in the pineapple shoot apical meristem, including early cellular vacuolation and separation of primitive leaves, with inflorescence development becoming evident around 15 days later (Liu et al., 2011; Espinosa et al., 2017).

From a molecular mechanistic perspective, ethylene plays a role in pineapple flowering by activating genes that are key to ethylene biosynthesis (Trusov and Botella, 2006; Liu and Fan, 2016; Espinosa et al., 2017; Zhang et al., 2021). Aminocyclopropane carboxylic acid (*ACC*), ACC synthase (*ACS*) and ACC oxidase (*ACO*) are key regulatory genes in the biosynthesis of ethylene. Genes encoding these enzymes have been shown to be key regulators of ethylene biosynthesis (Kende, 1993; Yang and Hoffman, 2003). In pineapples, *AcACS2* and *AcACO1* are significantly upregulated after ethephon treatment. Reducing the synthesis of endogenous *AcACS2* synthase through co-suppression technology decreases ethylene production in plants, and significantly delays flowering. The synthesis has been confirmed as a key gene in ethylene-induced flowering (Trusov and Botella, 2006; Liu and Fan, 2016; Espinosa et al., 2017). Meanwhile, the ethylene receptor genes *AcERS1b* and *AcETR2a*, as well as the ethylene response factor (*ERF*) genes, are also involved in ethylene signalling, collectively mediating the flowering transition (Liu et al., 2016; Zhang et al., 2021). Members of the ERF subfamily, especially Groups B2 and B4, are rapidly upregulated after ethephon treatment. Their promoter regions are enriched with ethylene responsive elements, enabling a direct response to ethylene signals and the regulation of downstream flowering related genes (Zhang et al., 2021). Furthermore, the *ERF* and *DREB* subfamilies are involved in the differentiation of floral organs. For example, *AcAP2/ERF21* and *AcAP2/ERF33* are highly expressed in sepals, whereas *AcAP2/ERF76* is specifically expressed in stamens. This suggests that they may play a crucial role in the initial phase of ethylene induced flowering in pineapple (Zhang et al., 2021).

Apart from ethylene, other plant hormones regulate the flowering process of pineapples by acting either synergistically or antagonistically with ethylene (Espinosa et al., 2017). Abscisic acid (ABA) and the cytochrome B-type hormone 2-isopentenyladenine (2-iP) promote pineapple flowering. Studies suggest that increased concentrations of ABA and 2-iP in the white tissue at the base of pineapple leave prompt the transition from vegetative growth to inflorescence initiation (Espinosa et al., 2017; Liu et al., 2021). By contrast, gibberellins (GA3) inhibit pineapple flowering (Espinosa et al., 2017). Reducing GA3 levels in the white tissue at the leaf base is crucial for the flowering transition to occur. Furthermore, applying ethephon can reduce GA3 levels over a prolonged period, thus promoting flowering (Espinosa et al., 2017). This contrasts with the mechanism in *Arabidopsis thaliana* whereby ethylene suppresses flowering by reducing GA levels and enhancing the accumulation of DELLAS proteins (Achard et al., 2007). Additionally, indole-3-acetic acid (IAA) and zeatin (ZT) exhibit inhibitory effects on pineapple flowering. Reduced levels of IAA and ZT in the white tissue at the leaf base also promote the transition to reproductive growth (Liu et al., 2011). Significant differences in ethylene sensitivity exist among different pineapple cultivars (Turnbull et al., 1999; Liu et al., 2018). For example, ‘Queen’ cultivars are susceptible to ethylene induced flowering, whereas ‘Smooth Cayenne’, ‘MD-2’, ‘Tainung No. 17’ and ‘Tainung No. 18’ cultivars are insensitive to ethylene induction (Lin et al., 2009; Liu et al., 2018). Under ethephon induced flowering, only some buds complete the transition from vegetative to reproductive growth (Lin et al., 2009; Liu et al., 2018).

Overall, the hormonal regulation of pineapple flowering is centred on ethylene as the core trigger signal, achieved through changes in the content and interactions of promotive hormones such as ABA and 2-iP, and inhibitory hormones such as GA3, IAA, and ZT. Additionally, cultivar specificity leads to significant variations in the effectiveness of ethylene induction.

#### 4.2.4 Plant intrinsic characteristics

Although natural flowering is significantly influenced by environmental factors, the plant’s condition is still a prerequisite for normal flowering. Plants in good nutritional condition are better able to initiate flower bud differentiation than those whose growth is impeded by low temperatures, nutrient deficiencies or water stress (Sun et al., 2019; Liu et al., 2021). Similar to other crops, excessive vegetative growth reduces the plant’s sensitivity to flowering stimuli, thereby inhibiting or delaying pineapple flowering (Cheng et al., 2017; Wang et al., 2025). Therefore, maintaining a balance between vegetative and reproductive growth is crucial. Once plants have attained floral competence, taking measures to inhibit vegetative growth, such as reducing the supply of nutrients and water, exposing the plants to low temperatures and shortening the photoperiod, can stimulate flower bud differentiation (Riboni et al., 2014; Brightbill and Sung, 2022; Chirivì and Betti, 2023). Under normal circumstances, flowering induction is typically carried out 10–12 months after planting pineapples, when the plants are growing vigorously and are more responsive to flowering agents (Liu et al., 2021). However, if the plant undergoes an excessively prolonged growth period resulting in an excessive number of leaves, its responsiveness to flowering agents is significantly diminished, making flowering induction more difficult (Liu et al., 2021).

Additionally, there are significant differences existing in flowering efficiency (flowering rate and timing) among different pineapple propagation materials (Ddungu, 1973). Among them, the suckers exhibit the best flowering performance, followed by the slips, while the crowns show the poorest results (Ddungu, 1973). Field trials under non-irrigated conditions confirmed that flowering rates within a single growing season reached 40% for the suckers, 10% for the slips, and only 2% for the crowns (Ddungu, 1973). Furthermore, multiple classic studies have verified that the fruiting cycle of suckers, especially medium and large-sized ones, is significantly shorter than that of slips and crowns (Hoare, 1948; Ochse et al., 1961). The main mechanism underlying this difference is that the suckers complete their development during the vegetative growth stage of the parent plant and originate from subterranean buds. They possess higher maturity and accumulate more sufficient photosynthates when separated from the parent plant, which provides a material basis for flower bud differentiation and flowering initiation (Ochse et al., 1961). This further indicates that the physiological maturity and the amount of accumulated photosynthates of planting materials at the time of separation from the parent plant are the key factors affecting flowering efficiency.

### 4.3 Genes influencing pineapple flowering

The artificial application of ethephon is a common method of inducing flowering in pineapple production, yet the molecular regulatory mechanisms involved remain incompletely understood (Espinosa et al., 2017). Following the completion of pineapple genome sequencing in recent years, researchers have conducted indepth studies on multiple flowering related gene families and transcriptional metabolic networks. These studies have revealed a series of key genes and pathways that regulate pineapple flowering, providing a foundation for the elucidation of the flowering mechanism (Feng et al., 2024).

The regulation of pineapple flowering involves the coordinated action of multiple gene families, which collectively participate in flower induction and floral organ development by responding to hormonal signals, light signals, and environmental stresses (Espinosa et al., 2017). For example, the subtilisin like serine protease (*SBT*) gene family encodes serine proteases that play a role in plant growth, development, and defence (Siezen and Leunissen, 1997; Figueiredo et al., 2017). The pineapple genome contains 54 *AcoSBT* members, which are grouped into six subfamilies and are unevenly distributed across 25 chromosomes (Jin et al., 2021b). Among these, *AcoSBT1.12* localises to the plasma membrane. Overexpression of *AcoSBT1.12* in Arabidopsis significantly delays flowering. This is achieved by increasing the expression of the key flowering repressor gene, Flowering locus C (*FLC*), while decreasing the expression of the flowering inducer genes, Flowering locus T (*FT*) and Suppressor of overexpression of constans 1 (*SOC1*) (Jin et al., 2021a). Phosphatidylethanolamine binding proteins (*PEBPs*) play a crucial role in regulating plant flowering time and morphogenesis (Jin et al., 2021a; Khosa et al., 2021; Zhu et al., 2021; Colleoni et al., 2024). The pineapple genome contains 11 *PEBP* family members, which are classified into three subfamilies based on phylogenetic relationships: FT-like, TFL-like, and MFT-like. Overexpression of *AcFT4* in Arabidopsis advances flowering by 6–7 days (Zhang et al., 2023).

The *BBX* transcription factor gene family encodes transcription factors containing the B-box domain that participate in plant photomorphogenesis, flowering time regulation and stress responses (Almada et al., 2009; Bai et al., 2016; An et al., 2019, 2021). A total of 19 *BBX* family members were identified in the pineapple genome. Most of them show high expression during flowering (Ouyang et al., 2022a). Specifically, *AcBBX5* suppresses flowering in Arabidopsis by binding to the promoter of the flowering promoting gene *AcFT* and inhibiting its expression (Ouyang et al., 2022b). Conversely, overexpression of *AcBBX18* in *Arabidopsis thaliana* promotes flowering by upregulating the expression of genes such as FT and SOC1 (Ouyang et al., 2022b). Within the pineapple Basic helix-loop-helix (*BHLH*) transcription factor family, AcCIB2 negatively regulates the flowering process. Overexpression of *AcCIB2* restores the early flowering phenotype in Arabidopsis *cib2* mutants (Aslam et al., 2020).

Furthermore, the MADS-box gene family is a core transcription factor family that regulates plant flowering and participates in the processes of floral organ identity determination and flowering time regulation (Takahiro and Hirano, 2006; Castelán-Muñoz et al., 2019; Adhikari and Kasahara, 2024; Zhang et al., 2024). Previous studies indicate that MADS-box genes in pineapple may regulate floral transition by integrating photoperiod and hormone signals. Genes related to the ABCDE model maybe involve in floral organ development and play crucial regulatory roles in the formation and development of organs, such as sepals and petals during pineapple flowering (Zhang et al., 2020; Hu et al., 2021; Pan et al., 2022). The B3 superfamily (*B3s*) is a plant specific group of transcription factors involved in processes such as embryonic development, hormone signalling and flowering regulation (Swaminathan et al., 2008; Sun et al., 2015). A total of 57 *AcB3* genes were identified in the pineapple genome (Ruan et al., 2021). Analysis of cis-acting elements revealed that *AcB3* gene promoters contain numerous hormone responsive elements for abscisic acid, ethylene and jasmonic acid, as well as light and stress responsive elements. Furthermore, the transcription levels of most of these genes are increased by ethylene induction, suggesting that they may be involved in ethylene induced flowering in pineapple (Ruan et al., 2021).

### 4.4 Technologies influencing pineapple flowering

The flowering time of pineapple is a key factor affecting the benefits of the pineapple industry. Scientifically understanding the underlying causes and implementing targeted flowering promotion or inhibition technologies is of great significance for avoiding losses caused by premature flowering and achieving fruiting period regulation (Bartholomew, 2014).

#### 4.4.1 Technologies for inducing pineapple flowering

##### 4.4.1.1 Ethephon

Ethephon is currently the most widely used flowering inducing agent for pineapple in production (Bartholomew, 2014). Following application of ethephon, pineapples decompose to produce ethylene. As an endogenous hormone capable of directly initiating reproductive growth in pineapples, ethylene accelerates the induction of flowering (Olsen et al., 2015; Van de Poel et al., 2015; Iqbal et al., 2017). Ethephon offers advantages such as low cost, simple application, and environmental safety, while significant differences exist in the sensitivity to ethephon induced flowering among different pineapple cultivars (**Table 1**).

**Table 1:** Comparison of agents and selection principles.

Agent	Core advantages	Applicable scenarios	Potential disadvantages
Calcium Carbide	Mild acting; effective for hard to flower cultivars	High temperature summer conditions; hard to flower cultivars	Flammable and explosive; complex operation
Ethephon	High efficiency, convenient application; low cost	Easy to flower cultivars; large scale cultivation	Prone to resulting in smaller fruits under high temperatures

Different pineapple cultivars exhibit highly variable responses to ethephon (Zhang et al., 2018) (**Table 2**). ‘Comte de Paris’ is classified as a sensitive cultivar, capable of inducing flowering at low concentrations of ethephon. In contrast, ‘Tainung No. 16’ is a less responsive cultivar that requires higher concentrations of ethephon to achieve optimal flowering induction (Zhang et al., 2018). Pu et al. (2024) investigated the effects of ethephon on flowering and quality in five pineapple cultivars. They found that the optimal flowering concentration was 400 mg/L for ‘Comte de Paris’ and ‘TaiNung No. 4’, while ‘MD-2’ and ‘Tainung No. 21’ required 800 mg/L. Interestingly, even at high concentrations, the flowering rate for ‘Tai Nong 22’ remained at just 33.33% (**Table 2**).

**Table 2:** Flowering inducing schemes for different cultivar types.

Cultivar type	Representative cultivars	Flowering inducing scheme	Flowering rate	Quality indicators
Easy to flower	‘Tainung No. 4’, ‘Comte de Paris ‘	Ethephon 200–400 mg/L, one application	≥ 95%	Single fruit weight increased by 30%
Relatively easy to flower	‘Tainung No. 16’, ‘Tainung No. 21’ ‘MD-2’	Ethephon 400–800 mg/L, one application; supplementary application for vigorous plants	≥90%	Number of small fruits
Hard to flower	‘Tainung No. 17’, ‘Smooth Cayenne’	Calcium carbide 1% (heart drenching, two applications) + Ethephon 600 mg/L (one application)	≥90%	Prone to smaller fruits under high temperatures

The main reason for the differences in sensitivity to ethylene-induced flowering among pineapple cultivars lies in the differential expression of key genes within the ethylene signalling pathway (Liu et al., 2020; Zhang et al., 2018). Receptor genes, such as *AcERS1a* and *AcETR2a*, perceive ethylene signals and transmit them to the nucleus by downregulating constitutive triple response 1 (*AcCTR1*) and upregulating ethylene insensitive 2 (*AcEIN2*). This subsequently regulates the expression of ethylene insensitive 3 (*AcEIN3*) and ethylene response factor 1/3 (*AcERF1/3*), which ultimately initiates the expression of the key flowering genes *AcFT*. The regulatory efficiency of this pathway differs between the ‘Comte de Paris’ and ‘Tainung No. 16’ cultivars, resulting in divergent flowering performance (Zhang et al., 2018).

In practical applications, the concentration of ethephon needs to be adjusted according to the season. For example, treating ‘Tainung No. 16’ with 300–600 mg/L of ethephon via stem injection during high summer temperatures achieved flowering rates of 93.83–97.78%. This treatment also resulted in significant increases in soluble sugar and soluble solids content, as well as reduced total acidity (Shu et al., 2019; Liu et al., 2024). For the ‘Pearl’ pineapple cultivar, the optimal summer flowering induction concentration is 200 mg/L; higher concentrations (800 mg/L) result in a significant reduction in individual fruit weight and length, lowering the fruit shape index to 0.88 and negatively impacting marketability (Liu et al., 2009).

Furthermore, combining ethephon with other regulators can optimise flowering induction. For example, treating ‘Tainung No. 16’ with 200 mg/L paclobutrazol plus 300 mg/L ethephon reduced plant height, increased leaf length, decreased adventitious bud formation, mitigated nutrient competition and improved fruit quality (Liu et al., 2009). For the ‘MD-2’ variety, adding 0.04% calcium carbonate to the ethephon solution reduced the required dosage from 200 mg/L to 20 mg/L, while also significantly increased the number of small fruits (Liu et al., 2021). Furthermore, combining urea with ethephon significantly increased the flowering efficiency of ‘Tainung No. 4’ pineapples (Deng et al., 2024). However, urea supplementation had no such effect on the ‘ Tainung No. 16’ variety (Hua et al., 2009).

##### 4.4.1.2 Calcium carbide

Calcium carbide is a commonly used flowering inducer in production (Bartholomew, 2014). Its core mechanism of action involves reacting with water to produce acetylene gas, which induces pineapple flower bud differentiation by mimicking ethylene signals (Bartholomew, 2014). Compared to other agents, calcium carbide offers the advantages of being mild in action and delivering stable results for varieties resistant to flowering induction. It is particularly suitable for high temperature summer environments or for varieties sensitive to flowering induction signals (**Table 1**).

Lin et al. (2009) demonstrated that applying a 1.0% calcium carbide solution to the crown of ‘Tainung No. 17’ twice at 48 hour intervals produced a comparable flowering rate to that achieved with ethephon treatment. However, as calcium carbide releases acetylene over a shorter period than ethephon, multiple applications are required. The “Tai Nong No. 4” pineapple variety achieved a 100% flowering rate when treated with 1.25%–1.5% calcium carbide in two applications to the core. However, only 71% of the fruits were cylindrical, which is significantly lower than the 99.38% observed in the ethylene treatment group. Furthermore, the fruit’s vitamin C and soluble solids content were also lower than in the ethylene treated group (Deng et al., 2024).Additionally, calcium carbide-induced flowering is significantly affected by environmental conditions and must be applied at night when temperatures remain below 25 °C (Deng et al., 2024). Calcium carbide also poses multiple safety issues and environmental hazards. It may contain toxic contaminants such as arsenic and phosphine, and it is prone to explosion when exposed to open flames. The acetylene gas produced during the reaction can also disrupt the pH balance of the soil, hindering sustainable production practices and posing risks to food safety (Onaha et al., 2007). Consequently, many regions including India have banned its use in commercial fruit production. Its application is generally discouraged for home growers due to food safety concerns. In China, purchasing calcium carbide requires stringent safety approvals and involves complex procedures. Given these issues, ethephon has now been widely adopted as a replacement flowering agent for calcium carbide in production (Deng et al., 2024).

##### 4.4.1.3 Other technologies

Besides ethephon and calcium carbide, cold water and naphthaleneacetic acid (NAA) may also be employed for pineapple flowering induction, though with less pronounced efficacy. Studies on ‘Tainung No. 16’ indicate that flowering rates following 2–3 applications of cold water irrigation ranged from 75.55% to 81.11%, significantly lower than the over 93% achieved with ethephon treatment (Liu et al., 2024). Moreover, ice water treatment resulted in markedly increased plant height, leaf width, and suckering in pineapple plants (Liu et al., 2024). Flowering rates for 20 mg/L NAA treatment reached only 56.79%, with no advantage in fruit quality, thus serving merely as an auxiliary method (Liu et al., 2024).

#### 4.4.2 Techniques for suppressing flowering in pineapples

Pineapple flowering suppression primarily addresses the issue of production season disruption caused by natural flowering (Bartholomew, 2014). The core approach involves using regulatory agents to reduce unintended flowering. Current research focuses on the application of ethylene synthesis inhibitors and growth regulators (Wang et al., 2007; Bartholomew et al., 2011; Bartholomew, 2014).

AminoethoxyVinylGlycin (AVG) is a known inhibitor of ACS activity which converts S-adenosylmethionine (SAM), which is the major methyl group donor for numerous transmethylation reactions to ACC, the immediate precursor of ethylene; it is the rate limiting enzyme in ethylene biosynthesis (Adams and Yang, 1979; Yu and Yang, 1979; Peters and Crist-Estes, 1989).

AVG is the active ingredient of a new chemical that in field trials was shown to reduce fruit abscission and to improve fruit quality (Bregoli et al., 2002; Ziosi et al., 2006). For instance, in pineapple production, applying AVG at a concentration of 500 mg·L^−1^ every 10–15 days for 4–5 consecutive applications significantly reduced the natural flowering rate of ‘Tainung No. 17’ pineapples and extended their natural flowering period by approximately 7 weeks (Wang et al., 2007; Rabie et al., 2013). Pires et al. (2025) found that AVG application at 100 mg·L^−1^ prior to natural induction significantly suppressed the natural flowering rate in ‘Pérola’ and ‘Vitória’ pineapples, with the inhibitory effect increasing with concentration. AVG application prior to natural flowering does not affect fruit quality or yield, whereas application during flowering causes nearly all fruits to become malformed. Consequently, AVG application must occur before natural flowering commences (Bartholomew et al., 2011).

Silver thiosulphate (STS) is an antiethylene growth regulator that inhibits plant flower development by modulating the expression of ethylene response factors and the crosstalk regulation of hormones such as auxin, salicylic acid, jasmonic acid, and cytokinin (Veen, 1983; Atta-Aly et al., 1987; Liu et al., 2018; Kim et al., 2024). Application of 1 mg/L silver nitrate at 30 days intervals for three consecutive treatments reduced the natural flowering rate from 50% to 27%, inhibiting natural flowering in pineapples (Sun et al., 2019). However, other studies suggest that STS application failed to inhibit natural flowering in ‘Pérola’, ‘MD-2’, and ‘Vitória’ pineapple cultivars, indicating that the role of STS in suppressing natural flowering in pineapple remains to be further validated (Maia et al., 2019).

Additionally, growth regulators such as paclobutrazol and ethephon may also assist in inhibiting flowering (Liu et al., 2021, 2024). Following application of 200 mg/L paclobutrazol to ‘Tainung No. 16’, vegetative growth was enhanced while natural flowering rates decreased. When combined with ethephon, this treatment balanced plant architecture with fruit development (Liu et al., 2024).

## 5 Conclusion

The flowering of pineapples is regulated by both internal and external factors. Ethylene is the core triggering signal that interacts synergistically or antagonistically with other hormones. The key natural requirements for flowering are low temperatures, appropriate photoperiods and plant condition. In production, synchronised flowering can be achieved using promoters such as ethephon, while inhibitors such as AVG can prevent premature flowering. Uniform flowering is ultimately a vital prerequisite for high quality, high yield pineapple production. Future research should focus on elucidating variety specific mechanisms and key gene functions, as well as developing environmentally friendly regulatory technologies to optimise pineapple harvest timing and enhance industry profitability.
